# Breakfast Consumption in Low-Income Hispanic Elementary School-Aged Children: Associations with Anthropometric, Metabolic, and Dietary Parameters

**DOI:** 10.3390/nu12072038

**Published:** 2020-07-09

**Authors:** Matthew R. Jeans, Fiona M. Asigbee, Matthew J. Landry, Sarvenaz Vandyousefi, Reem Ghaddar, Heather J. Leidy, Jaimie N. Davis

**Affiliations:** 1 The University of Texas at Austin, Department of Nutritional Sciences, 200 W 24th Street, Stop A2700, Austin, TX 78712, USA; fiona.asigbee@utexas.edu (F.M.A.); matthewlandry@utexas.edu (M.J.L.); sarvenaz.vandyousefi@nyulangone.org (S.V.); reemghaddar94@gmail.com (R.G.); heather.leidy@austin.utexas.edu (H.J.L.); jaimie.davis@austin.utexas.edu (J.N.D.); 2 Department of Pediatrics, Dell Medical Center, The University of Texas at Austin, 1400 Barbara Jordan Blvd, Austin, TX 78723, USA

**Keywords:** breakfast consumption, breakfast composition, children, dietary intake, dietary quality, diet patterns, cardiometabolic outcomes, adiposity

## Abstract

Breakfast consumption is associated with lower obesity prevalence and cardiometabolic risk and higher dietary quality (DQ) in children. Low-income, Hispanic populations are disproportionately affected by obesity and cardiometabolic risks. This study examined the relationship between breakfast consumption groups (BCG) on anthropometric, metabolic, and dietary parameters in predominately low-income, Hispanic children from 16 Texas schools. Cross-sectional data were from TX Sprouts, a school-based gardening, nutrition, and cooking randomized controlled trial. Anthropometric measurements included height, weight, body mass index, body fat percent via bioelectrical impedance, waist circumference, and blood pressure. Metabolic parameters included fasting plasma glucose, insulin, glycated hemoglobin, cholesterol, and triglycerides. DQ and BCG were assessed via two 24-h dietary recalls. Multivariate multiple regression examined relationships between BCG and anthropometric, metabolic, and dietary parameters. This study included 671 students (mean age 9 years, 58% Hispanic, 54% female, 66% free/reduced lunch, 17% breakfast skippers). No relationships were observed between BCG and anthropometric or metabolic parameters. BCG had higher DQ; higher daily protein, total sugar, and added sugar intake; and lower daily fat intake. Skipping breakfast was associated with lower DQ; higher daily fat intake; and lower daily protein intake. Longitudinal research examining breakfast quality on cardiometabolic outcomes in low-income, Hispanic children is warranted.

## 1. Introduction

Obesity prevalence has nearly tripled since 1975, affecting 18.5% of children and adolescents in the U.S., with those of Hispanic origin disproportionately affected at 25.8% [1]. Breakfast consumption has been a target of ongoing research in both predicting and preventing overweight and obesity prevalence [2]. Metabolic and physiological benefits of breakfast consumption in children include improved lipid panels, glucose control, and blood pressure and decreased fasting insulin [3,4,5,6]. Breakfast consumption is associated with lower cardiometabolic risks [2], including dyslipidemia [7], and lower metabolic syndrome risk in children [4,5,8,9]. However, few studies have evaluated breakfast consumption on cardiometabolic risks in primarily low-income, Hispanic children.

Despite breakfast consumption being associated with improved health outcomes in children and adolescents [5,10,11,12,13,14,15,16,17,18], the International Breakfast Research Initiative reports that only one third to one half of older children (11–15 years of age) consume breakfast every day [19]. In addition, the prevalence of skipping breakfast has been shown to increase with age [20]. Children and adolescents skip breakfast more than any other meal [21,22], with one study showing higher prevalence of skipping in Hispanic youth (32%) when compared to Caucasian youth (19%) [22]. Primary reasons for skipping breakfast include financial constraints and having inaccessibility to appropriate breakfast foods [23]. Hispanic households have higher prevalence of food insecurity, which has been associated with increased obesity prevalence in Hispanic children [24,25]. Nonetheless, while evidence supports the daily consumption of breakfast, studies have found conflicting results to substantiate this evidence. Some studies have shown a null association between breakfast consumption and weight management while others have found a positive association [26,27,28,29,30]. Potential reasons for the conflicting findings could be attributed to the quality of foods consumed at breakfast and/or the influence of breakfast on the overall diet.

Breakfast consumption is associated with meeting dietary intake recommendations and having superior overall diet quality in children [5,31,32]. Independent of breakfast consumption, breakfast composition has been associated with varied dietary quality [31,33]. Measures of dietary quality included evaluation of micronutrients, specifically shortfall nutrients (i.e., vitamin E, calcium, magnesium, iron, and zinc), and use of the Nutrient Rich Foods Index and the USDA Healthy Eating Index 2015 (HEI-2015) [31,32,33]. While associated with higher overall diet quality, regular breakfast consumers display higher intakes of saturated fats [5], sweets [33], and flavored milk [33] at breakfast, potentially eliciting a negative effect on adiposity and weight management. These counterintuitive findings could be due to fortification of common breakfast foods (i.e., cereals, nutrition bars, etc.) that tend to be high in saturated fats and added sugars, while still providing vitamins and minerals that contribute to diet quality. Furthermore, Deshmukh-Taskar et al. posed that consumption of milk at breakfast contributed to increased calcium intake [31]. Similar to flavored milk, this could explain why higher overall diet quality is observed, regardless of its other negative qualities.

These inconsistencies highlight the complex interaction between breakfast consumption and body weight, metabolism, and dietary habits. The evaluation of breakfast consumption is limited in primarily low-income and Hispanic populations, especially children. This study aims to (1) assess the relationship between breakfast consumption (skipping, intermittent consumption, and regular consumption) on anthropometric and metabolic parameters; (2) assess the relationship between breakfast consumption on daily dietary intake; and (3) assess the relationship between varied breakfast consumption patterns (intermittent vs. regular) on breakfast dietary intake in primarily low-income, Hispanic elementary school-aged children. It was hypothesized that breakfast consumption would be related to lower cardiometabolic risks and higher daily dietary quality, and that regular breakfast consumption would be related to a higher-quality breakfast meal than intermittent breakfast consumption.

## 2. Materials and Methods

### 2.1. Study Design

This cross-sectional study used baseline data from TX Sprouts, a school-based cluster randomized controlled gardening, nutrition, and cooking intervention. The study design for the TX Sprouts intervention has been described in detail elsewhere [34]. TX Sprouts recruited 3rd–5th grade students and their parents from 16 elementary schools in the Greater Austin, TX, area. All schools had to meet the following inclusion criteria: (1) high proportion of Hispanic children (>50%); (2) high proportion of children enrolled in the free and reduced lunch (FRL) program (>50%); (3) location within 60 miles of the University of Texas at Austin campus; and (4) no pre-existing school garden or gardening program. The first 16 schools that met criteria and agreed to participate were randomly assigned to receive the intervention (*n* = 8 schools) or delayed intervention (*n* = 8 schools), serving as the control group. This trial was registered at ClinicalTrials.gov (NCT02668744).

### 2.2. Study Population

**Figure 1:**
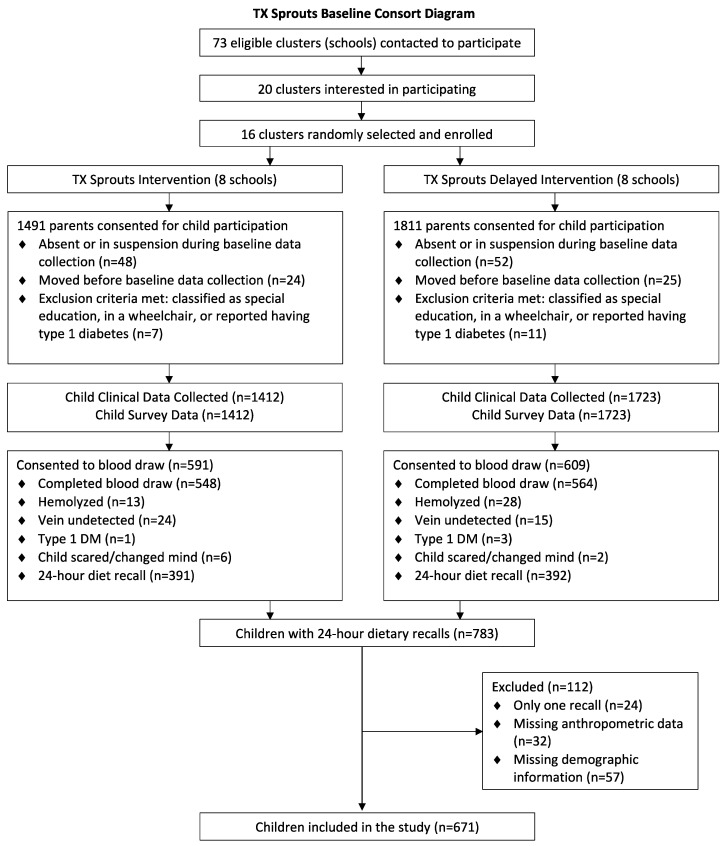
Consort diagram.

There was a total of 3302 students who obtained parental consent to participate in TX Sprouts. Of those, clinical data was collected on 3135. Sixteen students (eight male and eight female) were randomly selected from each grade level at each school to be contacted for recalls (*n* = 48/school). If any of the 16 originally selected students were unavailable or did not want to participate in recalls, then additional students were randomly selected to take their place. Dietary recalls were collected on a total subsample of 783 children—of which 23 had completed only one 24-h recall and were thus omitted. An additional child was omitted due to the breakfast energy cut point leaving only one day of recalls thereafter. Students were excluded from analyses for missing anthropometric data (*n* = 32) and demographic data (*n* = 56). In addition, one student was omitted due to the breakfast energy cut point. The total analytical sample was 671 students. Figure 1 provides a detailed consort diagram showing participant flow through the study.

### 2.3. Recruitment

All 3rd–5th grade students and parents of the recruited schools were contacted to participate via information tables at “Back to School” and “Meet the Teacher” events, flyers sent home with students, and classroom announcements made by teachers. Recruitment materials were available in both English and Spanish. Both parental consent and student assent were required for inclusion in the study. The study was conducted in accordance with the Declaration of Helsinki, and all procedures pertaining to human subjects were approved by the Institutional Review Boards of the University of Texas at Austin (IRB#2014-11-0045) and all associated school district review boards.

### 2.4. Anthropometric Parameters

Height was measured using a free-standing stadiometer to the nearest 0.1 cm (Seca, Birmingham, UK). In a private screening area, participants were asked to gather clothing above the waist so that waist circumference could be measured over skin using the National Health and Nutrition Examination Survey (NHANES) protocol [35]. Participants were asked to remove all footwear and heavy and/or layered clothing to obtain weight and bioelectrical impedance, which were assessed with a Tanita Body Fat Analyzer (Tanita Corporation of America Inc., IL, USA, model TBF 300) that was calibrated to −0.2 kg to account for clothing remaining. BMI z-scores were determined using Centers for Disease Control and Prevention (CDC) age- and gender-specific values [36]. Blood pressure was measured via an automated monitor (Omron, Schaumberg, IL, USA) with a child cuff or, in some cases, an adult cuff, which was used when the child cuff did not properly fit to provide an accurate reading.

### 2.5. Metabolic Parameters

Optional fasting blood draws were collected before the school day between 6:00 a.m. and 7:00 a.m. on a subsample of students at baseline. Those who opted to not participate in the blood draw were still able to participate in all other TX Sprouts evaluations and activities. Eligible students and their families received flyers and text message reminders about the optional blood draws and to come in fasting, having nothing to eat or drink other than water after midnight. Blood samples were collected by certified phlebotomists or nurses with experience drawing blood in children with obesity and were conducted in a private room at the schools. Students received a $20 incentive for participation in the blood draw. Samples were collected on site and transported on ice to the University of Texas at Austin laboratory.

Directly following collection, whole blood was placed on ice and transferred to the laboratory on the University of Texas at Austin campus, where it was spun and Clinical Laboratory Improvement Amendments (CLIA) certified glucose using HemoCue Glucose 201 (HemoCue America, Brea, CA, USA). Due to a larger than expected proportion of students having prediabetes using the American Diabetes Association definition (fasting plasma glucose of 100–125 mg/dL or HbA1c of 5.7–6.4%) [37], HbA1c measurement was added in the last two waves, explaining the lower number of samples and values observed for HbA1c. HbA1c assays using DCA Vantage Analyzer (Siemens Medical Solutions, Malvern, PA, USA) were performed on whole blood. Remaining blood was centrifuged, aliquoted, and stored at −80 °C. Samples were transported on dry ice to Baylor University for assessment of insulin, cholesterol, and triglycerides. Insulin was evaluated using an automated enzyme immunoassay system analyzer (Tosoh Bioscience, Inc. San Francisco, CA, USA). Total cholesterol, high-density lipoprotein (HDL), and triglyceride levels were measured using Vitros chemistry DT slides (Ortho Clinical Diagnostics Inc., Rochester, NY, USA); low-density lipoprotein (LDL) was calculated using the Friedwald equation [38].

### 2.6. Dietary Parameters

Dietary intake was collected using a validated two 24-h dietary recall method on a random subsample of children at baseline [39]. Recalls were collected via telephone by trained staff and supervised by a Registered Dietitian Nutritionist using Nutrition Data System for Research (NDS-R, 2016 version), a computer-based software application that facilitates the collection of recalls in a standardized fashion [40]. NDS-R generated nutrient and food/beverage servings and groupings, and HEI-2015 scores were calculated to assess dietary quality [41,42,43,44]. The HEI-2015 is composed of thirteen food components representative of the dietary recommendations based on the *Dietary Guidelines for Americans, 2015–2020*. These HEI-2015 components are divided into two groups: nine adequacy components (i.e., greens and beans, total fruits and vegetables, whole fruits, dairy, while grains, total protein foods, seafood and plant proteins, and fatty acids) and four moderation components (i.e., sodium, added sugars, refined grains, and saturated fat). Higher adequacy component scores are indicative of higher intake while higher moderation component scores are indicative of lower intake. The individual component scores are summed to an overall total HEI-2015 score ranging from 0 to 100. Higher HEI-2015 scores indicate higher dietary quality, per the *Dietary Guidelines for Americans, 2015–2020*.

Dietary intake data gathered by interview were governed by a multiple-pass interview approach [45]. Prior to the dietary recalls, Food Amounts Booklets, developed by the Nutrition Coordinating Center (NCC), were distributed to and sent home with the students. The booklets were provided in both English and Spanish and contained pictures of serving sizes to assist students in estimating serving sizes of foods and beverages reported during the dietary recall. Parents and/or guardians were allowed to assist with information regarding food items and portion sizes when needed. Students received a $10 incentive upon completion of both 24-h dietary recalls. Quality assurance was conducted on all dietary recall data by additional trained research staff. All daily dietary parameters were averages over the two days dietary recalls were conducted.

### 2.7. Breakfast Parameters

During each 24-h dietary recall, students were asked to name each eating occasion (EO) and the time of day when the EO occurred. Response options included: breakfast, brunch, lunch, dinner/supper, snack, beverage only, school lunch, or other. Dietary values were averaged across the two days of recall information to obtain mean values of consumption. Students were classified as breakfast consumers if they referred to an EO as “breakfast” and the energy intake was at least 15% of total daily energy and consumption occurred before 10:00 a.m. These criteria have been shown as an appropriate method for defining a breakfast meal [46,47,48,49]. If two breakfasts were consumed on the same day before 10:00 a.m. (i.e., one from home and one from school), then dietary values were combined before averaging with the second dietary recall.

In line with previous work evaluating breakfast consumption [31,50,51,52], breakfast consumption groups (BCG) were defined using the dietary recall data: (1) SKIPPERS, having no breakfast EO on either recall day; (2) INTERMITTENT, having a breakfast EO on only one recall day; and (3) REGULAR, having a breakfast EO on both recall days. As a result, breakfast dietary parameters for intermittent consumers were representative of intake for one day, while breakfast dietary parameters for regular consumers were representative of the average intake over two days.

### 2.8. Statistical Analysis

Data were examined for normality, and transformations were made if data deviated from normality. All variables were transformed for normality except BMI z-score, fasting plasma glucose, total cholesterol (mg/dL), non-HDL cholesterol (mg/dL), LDL cholesterol (mg/dL), HEI-2015 scores, carbohydrate (% kcal), fat (% kcal), total sugar (% kcal), and breakfast total sugar (% kcal). The negative reciprocal method was used for HbA1c (%) and diastolic BP (mmHg). The square root method was used for added sugar (% kcal), breakfast protein (% kcal), breakfast fat (% kcal), breakfast added sugar (% kcal), and whole grains (serving/day). The cubed method was used for BMI percentile (%). All other variables were log transformed. All analyses were performed using Stata version 16.0 (StataCorp, College Station, TX, USA), and the significance was set at *p* < 0.05.

Multivariate multiple regression assessed relationships between anthropometric and metabolic parameters by breakfast consumption groups. For anthropometric parameters, the model included waist circumference, percent body fat, BMI z-score, systolic blood pressure, and diastolic blood pressure. For metabolic parameters, one multivariate model included fasting glucose, insulin, total cholesterol, and triglycerides, and a second multivariate model included fasting glucose, insulin, HDL, non-HDL, LDL, and triglycerides. Since HbA1c was assessed only in the last two waves of the intervention, it was assessed in a third multivariate model including fasting glucose, total cholesterol, and triglycerides. All multivariate models pertaining to metabolic outcomes adjusted for age, sex, race/ethnicity, free and reduced lunch status, total energy, and BMI z-score (only in metabolic models).

Multivariate multiple regression assessed relationships between daily dietary parameters by breakfast consumption groups. Daily eating occasions, HEI-2015 scores, and total energy were assessed in univariate models. For daily nutrient intake, one multivariate model included percent protein, percent fat, percent carbohydrate, total fiber, and percent total sugar, and a second multivariate model included percent protein, percent fat, percent carbohydrate, soluble fiber, insoluble fiber, and percent added sugar. For daily food servings, one multivariate model included vegetables (including 100% juice and potatoes), fruit (including 100% juice), dairy (including flavored milk), SSBs (including flavored milk), whole grains, refined grains, meats, and legumes. A second multivariate model included vegetables (excluding 100% juice), fruit (excluding 100% juice), dairy (excluding flavored milk), SSBs (excluding flavored milk), whole grains, refined grains, meats, and legumes. A third multivariate model was the same as the second multivariate model but replaced vegetables (excluding 100% juice) with vegetables (excluding 100% juice and potatoes). All multivariate models pertaining to daily dietary parameters adjusted for age, sex, race/ethnicity, free and reduced lunch status, BMI z-score, day of the week, and total energy.

Multivariate multiple regression assessed relationships between breakfast composition between intermittent and regular consumers. Breakfast energy was assessed in a univariate model, adjusting for subsequent energy. For breakfast nutrient intake, one multivariate model included breakfast percent protein, percent fat, percent carbohydrate, total fiber, and percent total sugar, and a second multivariate model included breakfast percent protein, percent fat, percent carbohydrate, soluble fiber, insoluble fiber, and percent added sugar. For breakfast food servings, the multivariate model included breakfast whole and refined grain servings. All multivariate models pertaining to breakfast composition adjusted for age, sex, race/ethnicity, free and reduced lunch status, BMI z-score, day of the week, and total energy.

## 3. Results

### 3.1. Study Sample

The basic demographic, anthropometric, and dietary characteristics data are presented in Table 1 and Table 2. The sample was predominately Hispanic (58%) and female (54%), with an average age of 9.3 years. Reported enrollment of children in the Free and Reduced Lunch Program was 66%. The average BMI z-score was 0.8, and nearly 49% of children had overweight or obesity. The prevalence of BCG was 17% for skippers, 37% for intermittent consumers, and 46% for regular consumers. The average dietary quality, represented by the Healthy Eating Index—2015, was 53.8 out of a possible 100.

### 3.2. Relationships between Breakfast Consumption and Anthropometric and Metabolic Parameters

The relationships between BCG and anthropometric and metabolic parameters are presented in Table 3. There were no significant relationships between breakfast consumption groups and adiposity and metabolic parameters. A Pearson’s chi-square test was conducted to determine if these results could be a result of heterogeneous distributions of overweight and obese children between the BCG; however, the result was insignificant (*p* = 0.65) and showed homogenous distributions of BMI categories between the BCG.

### 3.3. Relationships between Breakfast Consumption and Daily Dietary Parameters

The relationships between BCG and daily dietary parameters are presented in Table 4. On average, there were fewer eating occasions observed in both SKIPPER and INTERMITTENT than REGULAR (β = −0.87 & β = −0.42, respectively; both *p* < 0.001). Differences in dietary quality, represented by total HEI-2015 scores, were detected between BCG, with SKIPPER and INTERMITTENT having lower scores than REGULAR (β = −3.88 & β = −2.69, respectively; both *p* < 0.02). Even with fewer eating occasions, INTERMITTENT had higher total daily energy than REGULAR (β = 174.73; *p* < 0.001). Daily macronutrient composition varied between BCG. Daily carbohydrate consumption was lower in SKIPPER and INTERMITTENT compared to REGULAR (β = −7.28 & β = −2.82, respectively; both *p* < 0.001). SKIPPER and INTERMITTENT had lower daily protein consumption than and REGULAR (β = −1.46 & β = −0.96, respectively; both *p* < 0.001). Lastly, SKIPPER consumed higher daily fat compared to REGULAR (β = 2.48; *p* = 0.001). Total fiber consumption was lower in both SKIPPER and INTERMITTENT than REGULAR (β = −1.03 & β = −0.98, respectively; both *p* < 0.01). Specifically, SKIPPER and INTERMITTENT had lower soluble fiber consumption than REGULAR (β = −0.62 & β = −0.39, respectively; both *p* < 0.001). Compared to REGULAR, both SKIPPER and INTERMITTENT had lower consumption of total sugar (β = −5.91 & β = −2.03, respectively; both *p* < 0.01). However, with added sugar, only SKIPPER had lower consumption than REGULAR (β = −1.64; both *p* = 0.002). Daily fruit consumption (including 100% juice) was lower in SKIPPER and INTERMITTENT compared to REGULAR (β = −0.43 & β = −0.24, respectively; both *p* < 0.01). However, when 100% juice was excluded, this relationship was attenuated. Daily consumption of whole grains was lower in SKIPPER compared to REGULAR (β = −0.14; *p* < 0.001).

### 3.4. Relationships between Breakfast Consumption and Breakfast Dietary Parameters

The relationships between BCG and breakfast dietary parameters are presented in Table 5. INTERMITTENT consumed lower energy at breakfast than REGULAR (β = −207.11; *p* < 0.001). Consumption of total fiber at breakfast was lower in INTERMITTENT compared to REGULAR (β = −1.75; *p* < 0.001). Specifically, both soluble and insoluble fiber consumption was lower in INTERMITTENT compared to REGULAR (β = −0.62 & β = −1.11, respectively; both *p* < 0.001). Consumption of whole grains at breakfast was lower in INTERMITTENT compared to REGULAR (β = −0.22; *p* < 0.001).

## 4. Discussion

Contrary to the hypothesis, this study evaluating low-income, Hispanic elementary school-aged children found no protective effects of breakfast consumption on adiposity and metabolic parameters. While regular breakfast consumption was linked to higher daily consumption of total and added sugar, it was also related to higher total HEI-2015 dietary quality scores; higher daily protein, carbohydrate, and fruit juice intake; and lower daily fat and energy intake. Furthermore, regular breakfast consumers had higher energy, fiber, and whole grain consumption in the breakfast meal compared to intermittent breakfast consumers. The link between breakfast consumption and both unhealthy and healthy dietary intake may explain the null effects of breakfast on adiposity and metabolic outcomes.

Similar to the results in this study, other recent studies have shown null or positive associations between breakfast consumption and obesity prevalence [26,27,28,29,30]. Fayet-Moore et al. showed breakfast consumption to be associated with lower overweight and obesity prevalence in children [13]. However, one year later, Fayet-Moore et al. examined breakfast consumption and breakfast cereal choice on anthropometric parameters in a similar cohort and observed no associations between breakfast consumption and overweight and obesity prevalence [28]. A longitudinal study showed increased breakfast consumption to be associated with higher obesity incidence and prevalence following an intervention spanning two and a half years [29]. These findings suggest that quantity and quality of foods consumed at breakfast may play a vital role in contributing to the potential cardiometabolic benefits associated with breakfast consumption.

Comparing breakfast consumption groups, skippers and intermittent breakfast consumers had less eating occasions, on average. However, this was to be expected as these groups are compared to those consistently consuming one main meal of the day. Both skippers and intermittent breakfast consumers had lower HEI-2015 scores than regular breakfast consumers. The USDA reports that children (6–17 years of age) have an average HEI-2015 score of 53 out of 100, which is the lowest of all other age groups [53]. The average HEI-2015 score in the current study was 53.8, similar to the national average for children in this age range [53]. Higher dietary quality was observed in regular consumers, with an HEI-2015 score approximately 3.9 and 2.7 points higher than skippers and intermittent consumers, respectively. These results are consistent with other studies showing that those who consume breakfast have higher diet quality [5,28,30,54,55].

Aside from overall dietary quality, those who regularly consumed breakfast had lower daily intake of fat and higher daily intake of protein, factors that are typically associated with reducing adiposity [56,57,58]. Though intermittent consumers had a lower number of eating occasions, this group managed to consume approximately 175 kilocalories more than regular breakfast consumers on a daily basis. Breakfast consumption has been associated with higher daily energy [55], but regular breakfast consumers still had lower daily energy intake than intermittent consumers. Regular breakfast consumers had higher daily servings of whole grains than skippers, likely contributing to the higher total and soluble fiber consumption observed. Literature supports that whole grain consumption is associated with decreased adiposity as well [59]. Though regular breakfast consumers had higher daily intake of protein and whole grains and lower daily intake of fat, this group also had higher daily intake of carbohydrates, total sugar, and fruit (including 100% juice) than both breakfast skippers and intermittent consumers. In addition, regular breakfast consumers had higher daily intake of added sugar than breakfast skippers. The relationships between fruit was attenuated when excluding 100% juice, suggesting that breakfast consumption of those beverages was driving the relationships observed with daily carbohydrate and total sugar intake. A similar trend was observed with dairy (including flavored milk), where lower consumption was observed in skippers than regular breakfast consumers. It is well-established that sugar consumption is positively associated with adiposity and blood glucose [60,61,62,63] and that whole fruit consumption leads to a greater reduction in hunger than consuming the same amount in fruit juice, with soluble fiber serving a prominent role [64,65]. Flood-Obbagy et al. showed that whole fruit consumption increased satiety more than fruit, fruit juice, and fruit juice with fiber, independent of energy density or fiber content [66]. Despite regular breakfast consumers having higher daily consumption of fruit juice than skippers and intermittent consumers, daily soluble fiber consumption remained superior. These results suggest that replacing fruit juice consumption with whole fruit consumption could further increase dietary quality and, in turn, improve cardiometabolic outcomes. Barr et al. reported higher sugar intake as dietary quality increased in breakfast consumers but suggested this could be due to higher fruit intakes observed [55]. Daily consumption of 100% fruit juice was driving the relationship with fruit in this study while contributing to total and added sugar consumption. Thus, it is possible that higher consumption of fruit juice and added sugar from other dietary sources resulted in deleterious effects of beneficial dietary intake, such as higher protein and whole grain consumption, contributing to the null results of breakfast consumption on anthropometric and metabolic parameters.

Relationships were observed in breakfast dietary parameters between regular and intermittent consumers. Intermittent consumers had lower energy, total fiber, soluble fiber, insoluble fiber, and whole grain consumption at breakfast compared to regular consumers. It is reported that Hispanic children (6–11 years of age) consume approximately 23% of energy at breakfast [67]. This study showed regular breakfast consumers had approximately 184 kilocalories more at breakfast than intermittent breakfast consumers, which is a difference of approximately 10–13% of daily energy for children of this age [68]. Daily energy was higher in intermittent consumers, so this is likely due to consumption of foods at other eating occasions throughout the day. In children, energy intake at breakfast has been shown to be homogenous across tertiles of dietary quality [55]. This compounded with the magnitude of caloric difference observed in the present study highlights the importance of promoting consumption of high-quality breakfast meals. In addition, previous work has shown intakes of carbohydrates and sugars at breakfast to be higher relative to breakfast energy [32]. Relationships between breakfast macronutrient consumption and breakfast sugar consumption were not observed between regular and intermittent consumers, however. While intermittent breakfast consumption was linked to lower soluble fiber intake at breakfast than regular breakfast consumption, the difference is relatively small at 0.62 g. Lower total fiber, soluble fiber, and insoluble fiber intake at breakfast was observed in intermittent consumers compared to regular consumers. Of the 1.75 g lower of total fiber, majority was insoluble fiber, which contributed 1.11 g. It can be postulated that the lower whole grain intake of 0.22 servings at breakfast observed in intermittent consumers contributed to these fiber results. However, the differences in fiber and whole grain content at breakfast were relatively small and lend little interpretation. The results observed propose dietary patterns surrounding breakfast consumption in this population may only differ primarily in energy content.

While both breakfast skippers and intermittent consumers had lower daily sugar intake than regular consumers, only breakfast skippers had lower daily added sugar intake than regular consumers. Added sugar was represented as percent of daily energy in this study. The insignificance of daily added sugar between intermittent and regular consumers highlights that, regardless of the higher daily energy observed, intermittent consumers had a similar percentage of added sugar intake to that of regular consumers. Sugar consumption has been studied as a food addiction [69]. Looking at food addiction in children with overweight, Filgueiras et al. showed that 95% of children (*n* = 139) showed at least one sign of food addiction, with 24% being diagnosed with food addiction and higher added sugar and ultra-processed food consumption as main contributors to food addiction [70]. The present study showed that breakfast consumers had higher daily consumption of total sugars, added sugars, and carbohydrates. These nutrients have properties of addiction, and consumption of breakfast meals composed of higher added sugar has been associated with higher added sugar consumption throughout the day [71]. As a result, this study posits a possible rationale for null relationships observed between breakfast consumption and cardiometabolic outcomes, suggesting that breakfast quality should be addressed in future interventions and guidelines.

### Limitations and Strengths

The current study had some limitations for consideration. First, the study was cross-sectional, thus causality cannot be inferred. This data, however, was baseline data from a randomized controlled trial. Therefore, future analyses will examine interventional effects of breakfast consumption on cardiometabolic outcomes and dietary intake in this population. The sample is predominantly Hispanic, so we were unable to distinguish any breakfast patterns or dietary composition by race or ethnicity. In addition, nearly 49% of children in this sample are classified as having overweight or obesity, so the sample is rather homogenous, and breakfast may not have a robust effect to elicit a response in our population. However, given the higher prevalence rates of obesity in Hispanic youth [1], it is important to examine dietary behaviors that are linked to obesity in this high-risk homogenous population. Another limitation to the current study is that there is no standard definition of breakfast, especially in the context of children, so it is possible that some of these results could be due to the chosen definition. The *Dietary Guidelines for Americans, 2015–2020* does not contain a standardized definition or recommendation for breakfast [68]. The energy cut point of 15% daily energy was chosen to exclude meals that were very low or no energy foods and beverages, i.e., a glass of water, single banana, nutrition bar, etc. Furthermore, the recommended amount of energy to be consumed at breakfast is dependent on the total number of EOs throughout the day [47]. Due to the lower number of EOs observed in Hispanic children (6–11 years of age) from the *What We Eat in America* data tables, the lower end of 15% daily energy proved appropriate for the breakfast definition in this study and has been recommended as the minimum energy requirement [47,72]. The recommendation for the breakfast energy threshold is 25%, but Hispanic children (6–11 years of age) consume, on average, 23% of daily energy at breakfast, so an upper limit of 25% did not seem appropriate [47,67].

One notable strength of the study is that dietary recalls were collected for two days, permitting the use of a definitive measure of dietary intake and evaluation of breakfast composition while controlling for confounding dietary variables. In turn, this study was able to discern and examine three breakfast consumption patterns (skipper, intermittent, and regular) instead of only two (eaters and skippers) seen in other studies. Dietary recall methodology allowed this study to examine different patterns of breakfast consumption on breakfast composition. One limitation, however, is that two dietary recalls may not be indicative of regular dietary behaviors.

## 5. Conclusions

Breakfast consumption was not associated with lower adiposity or healthier metabolic parameters, but regular breakfast consumption was linked to higher total HEI-2015 dietary quality scores; higher daily consumption of protein, carbohydrates, fruit juice, and whole grains; lower daily consumption of energy and dietary fat; and higher consumption of energy, fiber, and whole grains at breakfast. However, this study proposes that higher sugar intake of breakfast consumers played a role in masking the relationship with breakfast on adiposity and metabolic parameters. The results suggest that quality of foods consumed at breakfast plays a pivotal role in whether or not benefits of breakfast consumption are received.

## Figures and Tables

**Table 1:** Physical characteristics of the analytic sample (*n* = 671).

Variable	Value ^a^
Sex (F)	364 (54.3%)
Age (years)	9.3 (9.2, 9.3)
Ethnicity	
Hispanic	392 (58.4%)
Non-Hispanic	279 (41.6%)
Free/Reduced Lunch	446 (66.5%)
Breakfast Consumption Groups	
Skippers	114 (17.0%)
Intermittent Consumers	249 (37.1%)
Regular Consumers	308 (45.9%)
Height (cm)	138.4 (137.8, 139.1)
Weight (kg)	39.4 (38.5, 40.3)
BMI z-score	0.8 (0.8, 0.9)
BMI categories	
Overweight	129 (19.2%)
Obese	198 (29.5%)

^a^ All values are *n* (%) or mean (95% CI).

**Table 2:** Dietary characteristics of the analytic sample (*n* = 671).

Variable	Mean ^a^	95% CIs
Eating occasions	3.3	(3.3, 3.4)
Healthy Eating Index-2015	53.8	(52.8, 54.7)
Total energy (kcal/day)	1446.2	(1405.1, 1487.3)
Carbohydrate (% kcal)	49.7	(49.0, 50.3)
Protein (% kcal)	16.2	(15.9, 16.5)
Fat (% kcal)	33.3	(32.8, 33.8)
Total fiber (g)	12.1	(11.7, 12.6)
Soluble fiber (g)	3.8	(3.7, 4.0)
Insoluble fiber (g)	8.2	(7.9, 8.5)
Total sugar (% kcal)	20.7	(20.2, 21.3)
Added sugar (% kcal)	10.1	(9.6,10.5)
Total vegetables (serving/day) ^b^	1.7	(1.6, 1.8)
Excluding 100% juice	1.7	(1.6, 1.8)
Excluding 100% juice and potatoes	1.5	(1.4, 1.6)
Total Fruits (serving/day)	1.5	(1.4, 1.7)
Excluding 100% juice	0.9	(0.8, 1.0)
Dairy (serving/day)	1.7	(1.6, 1.8)
Excluding flavored milk	1.5	(1.4, 1.6)
Sugar-sweetened beverages (serving/day)	0.7	(0.7, 0.8)
Excluding flavored milk	0.5	(0.4, 0.6)
Whole grains (serving/day)	1.1	(1.0, 1.2)
Refined grains (serving/day)	4.6	(4.4, 4.9)
Meat (serving/day)	3.7	(3.5, 3.8)
Legumes (serving/day)	0.2	(0.16, 0.23)

^a^ Dietary data reflects the average of two days. ^b^ Servings per day.

**Table 3:** Multivariate regression models of anthropometric and metabolic parameters with regular consumers as the referent group.

	Overall ^†^	<-------------------- Skipper -------------------->			<-------------------- Intermittent -------------------->		
Variable	*p*	β	(95% CI)	*p*	β	(95% CI)	*p*
*Anthropometric Parameters (n = 671)*							
Waist circumference (cm) ^a^	0.09	0.59	(−1.98, 3.16)	0.62	2.09	(0.08, 4.10)	0.03
Total body fat (%) ^a^	0.39	−0.05	(−1.95, 1.86)	0.76	0.82	(−0.67, 2.31)	0.17
BMI z-score ^a^	0.12	0.12	(−0.12, 0.36)	0.33	0.19	(0.01, 0.38)	0.04
Systolic blood pressure (mmHg) ^a^	0.58	−0.10	(−2.51, 2.31)	0.99	0.84	(−1.05, 2.73)	0.33
Diastolic blood pressure (mmHg) ^a^	0.72	0.63	(−1.46, 2.72)	0.64	0.43	(−1.21, 2.06)	0.43
*Metabolic Parameters (n = 344)*							
Fasting glucose (mg/dL) ^b,c,d^	0.89	0.47	(−2.23, 3.17)	0.73	−0.21	(−2.42, 2.01)	0.85
Insulin (µIU/mL) ^b,c,d^	0.67	0.89	(−2.29, 4.08)	0.39	1.26	(−1.35, 3.87)	0.90
Triglycerides (mg/dL) ^b,c,d^	0.37	4.14	(−7.46, 15.74)	0.61	−5.98	(−15.49, 3.52)	0.32
Total cholesterol (mg/dL) ^b,c,d^	0.95	0.42	(−6.82, 7.66)	0.91	0.93	(−5.00, 6.87)	0.76
HDL (mg/dL) ^c^	0.96	−0.30	(−2.95, 2.36)	0.89	−0.31	(−2.49, 1.87)	0.77
Non-HDL (mg/dL) ^c^	0.91	0.69	(−5.86, 7.24)	0.84	1.18	(−4.19, 6.54)	0.67
LDL (mg/dL) ^c^	0.54	−0.15	(−6.05, 5.74)	0.96	2.45	(−2.38, 7.28)	0.32
HbA1c (%) ^d^	0.49	−0.09	(−0.15, 0.09)	0.50	0.04	(−0.06, 0.15)	0.53

Abbreviations: BMI, body mass index; HbA1c, glycolated hemoglobin A1C; HDL, high-density lipoprotein; LDL, low-density lipoprotein. Multivariate regression assessed relationships between anthropometric parameters (*n* = 671) and metabolic parameters (*n* = 344) by breakfast consumption groups. Regular consumers served as the referent group for all analyses. ^†^ Overall effect of breakfast consumption groups. ^a^ Model included waist circumference, total body fat, BMI z-score, and blood pressure. ^b^ Model included fasting glucose, insulin, total cholesterol, and triglycerides. ^c^ Model included fasting glucose, insulin, triglycerides, HDL, non-HDL, and LDL. ^d^ HbA1c was assessed only in the last two waves of the intervention, indicative of the smaller sample size (*n* = 237), so it was assessed in an independent model including fasting glucose, insulin, total cholesterol, and triglycerides. A priori covariates included: age, sex, race/ethnicity, free/reduced lunch status, daily energy, and BMI z-score (for metabolic parameters).

**Table 4:** Multivariate regression models of daily nutrient intake and daily food and beverage servings with regular consumers as the referent group (*n* = 671).

	Overall ^†^	<----------------- Skipper ----------------->			<--------------- Intermittent --------------->		
Variable	*p*	β	(95% CI)	*p*	β	(95% CI)	*p*
*Dietary Intake Parameters*							
Eating occasions	<0.001 *	−0.87	(−1.01, −0.73)	<0.001 *	−0.42	(−0.53, −0.30)	<0.001 *
HEI-2015	<0.005 *	−3.88	(−6.52, −1.24)	0.004 *	−2.69	(−4.76, −0.62)	0.01 *
Total energy (kcal)	<0.004 *	111.35	(−5.11, 227.81)	0.14	174.73	(84.25, 265.20)	0.001 *
Protein (%kcal) ^a,b^	<0.001 *	−1.46	(−2.26, −0.66)	<0.001 *	−0.96	(−1.59, −0.34)	0.001 *
Fat (%kcal) ^a,b^	<0.005 *	2.48	(1.00, 3.95)	0.001 *	0.90	(−0.25, 2.06)	0.13
Carbohydrate (%kcal) ^a,b^	<0.001 *	−7.28	(−9.10, −5.46)	<0.001 *	−2.82	(−4.24, −1.39)	<0.001 *
Total fiber (g) ^a,b^	<0.007 *	−1.03	(−1.97, −0.09)	<0.02 *	−0.98	(−1.72, −0.25)	0.004 *
Total sugar (%kcal) ^a,b^	<0.001 *	−5.91	(−7.55, −4.26)	<0.001 *	−2.03	(−3.32, −0.75)	0.002 *
Soluble fiber (g) ^b^	<0.001 *	−0.62	(−0.93, −0.31)	<0.001 *	−0.39	(−0.63, −0.14)	0.001 *
Insoluble fiber (g) ^b^	0.051	−0.46	(−1.23, 0.30)	0.12	−0.61	(−1.21, −0.01)	0.02
Added sugar (%kcal) ^b^	0.003 *	−1.64	(−2.91, −0.37)	0.002 *	0.31	(−0.68, 1.30)	0.81
*Food and Beverage Servings*							
Whole grains (serving/day) ^c,d,e^	0.002 *	−0.14	(−0.40, 0.13)	0.001 *	−0.05	(−0.26, 0.16)	0.37
Refined grains (serving/day) ^c,d,e^	0.16	−0.21	(−0.69, 0.28)	0.06	−0.27	(−0.65, 0.11)	0.32
Meats (serving/day) ^c,d,e^	0.98	0.17	(−0.30, 0.65)	0.94	0.10	(−0.27, 0.48)	0.88
Legumes (serving/day) ^c,d,e^	0.07	0.10	(0.01, 0.19)	0.02	0.01	(−0.06, 0.08)	0.64
Vegetables, including 100% juice & potatoes (serving/day) ^c^	0.87	−0.001	(−0.26, 0.26)	0.86	−0.09	(−0.29, 0.12)	0.69
Fruit, including 100% juice (serving/day) ^c^	0.001 *	−0.43	(−0.77, −0.08)	0.002 *	−0.24	(−0.51, 0.03)	0.003 *
Dairy, including flavored milk (serving/day) ^c^	0.09	−0.18	(−0.40, 0.03)	0.03	−0.09	(−0.26, 0.08)	0.30
SSBs, including flavored milk (serving/day) ^c^	0.44	−0.09	(−0.28, 0.09)	0.42	0.10	(−0.04, 0.25)	0.52
Vegetables, excluding 100% juice (serving/day) ^d,e^	0.85	0.0004	(−0.26, 0.26)	0.85	−0.09	(−0.26, 0.26)	0.66
Fruit, excluding 100% juice (serving/day) ^d,e^	0.29	−0.10	(−0.40, 0.19)	0.27	−0.05	(−0.28, 0.18)	0.15
Dairy, excluding flavored milk (serving/day) ^d,e^	0.40	−0.07	(−0.28, 0.14)	0.27	−0.09	(−0.28, 1.4)	0.26
SSBs, excluding flavored milk (serving/day) ^d,e^	0.65	0.02	(−0.14, 0.18)	0.61	0.10	(−0.03, 0.22)	0.37
Vegetables, excluding 100% juice & potatoes (serving/day) ^e^	0.57	0.03	(−0.22, 0.28)	0.66	−0.12	(−0.31, 0.07)	0.46

Abbreviations: HEI, healthy eating index; SSBs, sugar-sweetened beverages. * Indicates a statistically significant value of *p* < 0.05. Multivariate regression assessed relationships between all dietary parameters by breakfast consumption groups (*n* = 671). Regular consumers served as the referent group for all analyses. ^†^ Overall effect of breakfast consumption groups. Eating occasions, HEI-2015 scores, and total energy were assessed in univariate models. ^a^ Model included percent protein, percent fat, percent carbohydrate, total fiber, and percent total sugar. ^b^ Model included percent protein, percent fat, percent carbohydrate, soluble fiber, insoluble fiber, and percent added sugar. ^c^ Model included vegetables (including 100% juice and potatoes), fruit (including 100% juice), dairy (including flavored milk), SSBs (including flavored milk), whole grains, refined grains, meats, and legumes. ^d^ Model included vegetables (excluding 100% juice), fruit (excluding 100% juice), dairy (excluding flavored milk), SSBs (excluding flavored milk), whole grains, refined grains, meats, and legumes. ^e^ Model was the same as the second model, replacing vegetables (excluding 100% juice) with vegetables (excluding 100% juice and potatoes). *A priori* covariates included: age, sex, race/ethnicity, free/reduced lunch status, BMI z-score, day of the week, and total energy.

**Table 5:** Multivariate regression models of breakfast nutrient intake and breakfast food servings with regular consumers as the referent group (*n* = 557).

	Intermittent		
Variable	β	(95% CI)	*p*
*Breakfast Dietary Intake Parameters*			
Breakfast total energy (kcal)	−207.11	(−208.88, −159.76)	<0.001 *
Breakfast protein (%kcal) ^a,b^	−0.34	(−1.13, 0.44)	0.13
Breakfast fat (%kcal) ^a,b^	0.51	(−1.80, 2.82)	0.59
Breakfast carbohydrate (%kcal) ^a,b^	−0.37	(−3.24, 2.51)	0.43
Breakfast total fiber (g) ^a^	−1.75	(−2.07, −1.43)	<0.001 *
Breakfast total sugar (%kcal) ^a^	−0.61	(−3.12, 1.89)	0.63
Breakfast soluble fiber (g) ^b^	−0.62	(−0.74, −0.51)	<0.001 *
Breakfast insoluble fiber (g) ^b^	−1.11	(−1.34, −0.87)	<0.001 *
Breakfast added sugar (%kcal) ^b^	1.38	(−0.52, 3.29)	0.81
*Breakfast Food Servings*			
Breakfast whole grains (serving/day) ^c^	−0.22	(−0.31, −0.13)	<0.001 *
Breakfast refined grains (serving/day) ^c^	−0.03	(−0.12, 0.07)	0.14

* Indicates a statistically significant value of *p* < 0.05. Multivariate regression assessed relationships between all dietary parameters by breakfast consumption groups (*n* = 557). Regular consumers served as the referent group for all analyses. Overall effects not reported due to contrast of only two levels of breakfast consumption groups. Breakfast energy was assessed in a univariate model, adjusting for subsequent energy. ^a^ Model included breakfast percent protein, percent fat, percent carbohydrate, total fiber, and percent total sugar. ^b^ Model included breakfast percent protein, percent fat, percent carbohydrate, soluble fiber, insoluble fiber, and percent added sugar. ^c^ Model included breakfast whole and refined grain servings. *A priori* covariates included: age, sex, race/ethnicity, free/reduced lunch status, BMI z-score, day of the week, and total energy.

## References

[1] Hales C.M., Carroll M.D., Fryar C.D., Ogden C.L. (2017). Prevalence of obesity among adults and youth: United States, 2015–2016. NCHS Data Brief..

[2] Monzani A., Ricotti R., Caputo M., Solito A., Archero F., Bellone S., Prodam F. (2019). A systematic review of the association of skipping breakfast with weight and cardiometabolic risk factors in children and adolescents. What should we better investigate in the future?. Nutrients.

[3] Smith K.J., Gall S.L., McNaughton S.A., Blizzard L., Dwyer T., Venn A.J. (2010). Skipping breakfast: Longitudinal associations with cardiometabolic risk factors in the Childhood determinants of adult health study. Am. J. Clin. Nutr..

[4] Shafiee G., Kelishadi R., Qorbani M., Motlagh M.E., Taheri M., Ardalan G., Taslimi M., Poursafa P., Heshmat R., Larijani B. (2013). Association of breakfast intake with cardiometabolic risk factors. J. Pediatr..

[5] Ho C.Y., Huang Y.C., Lo Y.T., Wahlqvist M.L., Lee M.S. (2015). Breakfast is associated with the metabolic syndrome and school performance among Taiwanese children. Res. Dev. Disabil..

[6] Bauer L.B., Reynolds L.J., Douglas S.M., Kearney M.L., Hoertel H.A., Shafer R.S., Thyfault J.P., Leidy H.J. (2015). A pilot study examining the effects of consuming a high-protein vs normal-protein breakfast on free-living glycemic control in overweight/obese ‘breakfast skipping’ adolescents. Int. J. Obes..

[7] Deshmukh-Taskar P., Nicklas T.A., Radcliffe J.D., O’Neil C.E., Liu Y. (2013). The relationship of breakfast skipping and type of breakfast consumed with overweight/obesity, abdominal obesity, other cardiometabolic risk factors and the metabolic syndrome in young adults. The National Health and Nutrition Examination Survey (NHANES): 1999–2006. Public Health Nutr..

[8] Monzani A., Rapa A., Fuiano N., Diddi G., Prodam F., Bellone S., Bona G. (2014). Metabolic syndrome is strictly associated with parental obesity beginning from childhood. Clin. Endocrinol..

[9] Osawa H., Sugihara N., Ukiya T., Ishizuka Y., Birkhed D., Hasegawa M., Matsukubo T. (2015). Metabolic syndrome, lifestyle, and dental caries in Japanese school children. Bull. Tokyo Dent. Coll..

[10] O’Neil C.E., Nicklas T.A., Fulgoni V.L. (2015). Nutrient intake, diet quality, and weight measures in breakfast patterns consumed by children compared with breakfast skippers: NHANES 2001–2008. AIMS Public Health.

[11] Smetanina N., Albaviciute E., Babinska V., Karinauskiene L., Albertsson-Wikland K., Petrauskiene A., Verkauskiene R. (2015). Prevalence of overweight/obesity in relation to dietary habits and lifestyle among 7–17 years old children and adolescents in Lithuania. BMC Public Health.

[12] Zakrzewski J.K., Gillison F.B., Cumming S., Church T.S., Katzmarzyk P.T., Broyles S.T., Champagne C.M., Chaput J.P., Denstel K.D., Fogelholm M. (2015). Associations between breakfast frequency and adiposity indicators in children from 12 countries. Int. J. Obes. Suppl..

[13] Fayet-Moore F., Kim J., Sritharan N., Petocz P. (2016). Impact of breakfast skipping and breakfast choice on the nutrient intake and body mass index of australian children. Nutrients.

[14] Smith K.J., Breslin M.C., McNaughton S.A., Gall S.L., Blizzard L., Venn A.J. (2017). Skipping breakfast among Australian children and adolescents; findings from the 2011–12 national nutrition and physical activity survey. Aust. N. Z. J. Public Health.

[15] Gotthelf S.J., Tempestti C.P. (2017). Breakfast, nutritional status, and socioeconomic outcome measures among primary school students from the City of Salta: A cross-sectional study. Arch. Argent. Pediatr..

[16] Nilsen B.B., Yngve A., Monteagudo C., Tellstrom R., Scander H., Werner B. (2017). Reported habitual intake of breakfast and selected foods in relation to overweight status among seven- to nine-year-old Swedish children. Scand. J. Public Health.

[17] Tee E.S., Nurliyana A.R., Norimah A.K., Mohamed H., Tan S.Y., Appukutty M., Hopkins S., Thielecke F., Ong M.K., Ning C. (2018). Breakfast consumption among Malaysian primary and secondary school children and relationship with body weight status—Findings from the MyBreakfast Study. Asia Pac. J. Clin. Nutr..

[18] Archero F., Ricotti R., Solito A., Carrera D., Civello F., Di Bella R., Bellone S., Prodam F. (2018). Adherence to the mediterranean diet among school children and adolescents living in northern italy and unhealthy food behaviors associated to overweight. Nutrients.

[19] Gibney M.J., Barr S.I., Bellisle F., Drewnowski A., Fagt S., Livingstone B., Masset G., Varela Moreiras G., Moreno L.A., Smith J. (2018). Breakfast in human nutrition: The international breakfast research initiative. Nutrients.

[20] Currie C.Z.C., Morgan A., Currie D., de Looze M., Roberts C., Samdal O., Smith O.R.F., Barnekow V. (2012). Social determinants of health and well-being among young people. Health Behaviour in School-Aged Children (HBSC) Study: International Report from the 2009/2010 Survey.

[21] Murata M. (2000). Secular trends in growth and changes in eating patterns of Japanese children. Am. J. Clin. Nutr..

[22] Dwyer J.T., Evans M., Stone E.J., Feldman H.A., Lytle L., Hoelscher D., Johnson C., Zive M., Yang M. (2001). Child and adolescents′ eating patterns influence their nutrient intakes. J. Am. Diet. Assoc..

[23] Van Kleef E., Vingerhoeds M.H., Vrijhof M., van Trijp H.C.M. (2016). Breakfast barriers and opportunities for children living in a Dutch disadvantaged neighbourhood. Appetite.

[24] Papas M.A., Trabulsi J.C., Dahl A., Dominick G. (2016). Food insecurity increases the odds of obesity among young hispanic children. J. Immigr. Minor. Health.

[25] Potochnick S., Perreira K.M., Bravin J.I., Castaneda S.F., Daviglus M.L., Gallo L.C., Isasi C.R. (2019). Food insecurity among hispanic/latino youth: Who is at risk and what are the health correlates?. J. Adolesc. Health.

[26] Kuriyan R., Thomas T., Sumithra S., Lokesh D.P., Sheth N.R., Joy R., Bhat S., Kurpad A.V. (2012). Potential factors related to waist circumference in urban South Indian children. Indian Pediatr..

[27] Kupers L.K., de Pijper J.J., Sauer P.J., Stolk R.P., Corpeleijn E. (2014). Skipping breakfast and overweight in 2- and 5-year-old Dutch children-the GECKO Drenthe cohort. Int. J. Obes..

[28] Fayet-Moore F., McConnell A., Tuck K., Petocz P. (2017). Breakfast and breakfast cereal choice and its impact on nutrient and sugar intakes and anthropometric measures among a nationally representative sample of australian children and adolescents. Nutrients.

[29] Polonsky H.M., Bauer K.W., Fisher J.O., Davey A., Sherman S., Abel M.L., Hanlon A., Ruth K.J., Dale L.C., Foster G.D. (2019). Effect of a breakfast in the classroom initiative on obesity in urban school-aged children: A cluster randomized clinical trial. JAMA Pediatr..

[30] Coulthard J.D., Palla L., Pot G.K. (2017). Breakfast consumption and nutrient intakes in 4–18-year-olds: UK national diet and nutrition survey rolling programme (2008–2012). Br. J. Nutr..

[31] Deshmukh-Taskar P.R., Nicklas T.A., O’Neil C.E., Keast D.R., Radcliffe J.D., Cho S. (2010). The relationship of breakfast skipping and type of breakfast consumption with nutrient intake and weight status in children and adolescents: The National health and nutrition examination survey 1999–2006. J. Am. Diet. Assoc..

[32] Drewnowski A., Rehm C.D., Vieux F. (2018). Breakfast in the United States: Food and nutrient intakes in relation to diet quality in national health and examination survey 2011–2014. a study from the international breakfast research initiative. Nutrients.

[33] Lepicard E.M., Maillot M., Vieux F., Viltard M., Bonnet F. (2017). Quantitative and qualitative analysis of breakfast nutritional composition in French schoolchildren aged 9–11 years. J. Hum. Nutr. Diet..

[34] Davis J., Nikah K., Asigbee F.M., Landry M.J., Vandyousefi S., Ghaddar R., Hoover A., Jeans M., Pont S.J., Richards D. (2019). Design and participant characteristics of TX sprouts: A school-based cluster randomized gardening, nutrition, and cooking intervention. Contemp Clin. Trials.

[35] Centers for Disease Control and Prevention (2007). Anthropometry Procedures Manual. https://www.cdc.gov/nchs/data/nhanes/nhanes_07_08/manual_an.pdf.

[36] Centers for Disease Control and Prevention (2000). Clinical Growth Charts. https://www.cdc.gov/growthcharts/clinical_charts.htm.

[37] American Diabetes A. (2014). Diagnosis and classification of diabetes mellitus. Diabetes Care.

[38] Friedewald W.T., Levy R.I., Fredrickson D.S. (1972). Estimation of the concentration of low-density lipoprotein cholesterol in plasma, without use of the preparative ultracentrifuge. Clin. Chem..

[39] Burrows T.L., Martin R.J., Collins C.E. (2010). A systematic review of the validity of dietary assessment methods in children when compared with the method of doubly labeled water. J. Am. Diet. Assoc..

[40] Feskanich D., Sielaff B.H., Chong K., Buzzard I.M. (1989). Computerized collection and analysis of dietary intake information. Comput. Methods Programs Biomed..

[41] National Cancer Institute (2015). Developing the Healthy Eating Index. https://epi.grants.cancer.gov/hei/developing.html.

[42] Kirkpatrick S.I., Reedy J., Krebs-Smith S.M., Pannucci T.E., Subar A.F., Wilson M.M., Lerman J.L., Tooze J.A. (2018). Applications of the healthy eating index for surveillance, epidemiology, and intervention research: Considerations and caveats. J. Acad. Nutr. Diet..

[43] Krebs-Smith S.M., Pannucci T.E., Subar A.F., Kirkpatrick S.I., Lerman J.L., Tooze J.A., Wilson M.M., Reedy J. (2018). Update of the healthy eating index: HEI-2015. J. Acad. Nutr. Diet..

[44] Reedy J., Lerman J.L., Krebs-Smith S.M., Kirkpatrick S.I., Pannucci T.E., Wilson M.M., Subar A.F., Kahle L.L., Tooze J.A. (2018). Evaluation of the healthy eating index-2015. J. Acad. Nutr. Diet..

[45] Johnson R.K., Driscoll P., Goran M.I. (1996). Comparison of multiple-pass 24-hour recall estimates of energy intake with total energy expenditure determined by the doubly labeled water method in young children. J. Am. Diet. Assoc..

[46] Leech R.M., Worsley A., Timperio A., McNaughton S.A. (2015). Characterizing eating patterns: A comparison of eating occasion definitions. Am. J. Clin. Nutr..

[47] O′Neil C.E., Byrd-Bredbenner C., Hayes D., Jana L., Klinger S.E., Stephenson-Martin S. (2014). The role of breakfast in health: Definition and criteria for a quality breakfast. J. Acad. Nutr. Diet..

[48] Pereira M.A., Erickson E., McKee P., Schrankler K., Raatz S.K., Lytle L.A., Pellegrini A.D. (2011). Breakfast frequency and quality may affect glycemia and appetite in adults and children. J. Nutr..

[49] Siega-Riz A.M., Popkin B.M., Carson T. (1998). Trends in breakfast consumption for children in the United States from 1965–1991. Am. J. Clin. Nutr..

[50] Cho S., Dietrich M., Brown C.J., Clark C.A., Block G. (2003). The effect of breakfast type on total daily energy intake and body mass index: Results from the third national health and nutrition examination survey (NHANES III). J. Am. Coll. Nutr..

[51] Deshmukh-Taskar P.R., Radcliffe J.D., Liu Y., Nicklas T.A. (2010). Do breakfast skipping and breakfast type affect energy intake, nutrient intake, nutrient adequacy, and diet quality in young adults? NHANES 1999–2002. J. Am. Coll. Nutr..

[52] Lyerly J.E., Huber L.R., Warren-Findlow J., Racine E.F., Dmochowski J. (2014). Is breakfast skipping associated with physical activity among U.S. adolescents? A cross-sectional study of adolescents aged 12–19 years, National Health and Nutrition Examination Survey (NHANES). Public Health Nutr..

[53] United States Department of Agriculture (2016). HEI Scores for Americans. Average Healthy Eating Index-2015 Scores for Americans by Age Group, WWEIA/NHANES 2015–2016. https://fns-azureedge.net/sites/default/files/media/file/HEI-2015_1516_web.pdf.

[54] Gaal S., Kerr M.A., Ward M., McNulty H., Livingstone M.B.E. (2018). Breakfast consumption in the UK: Patterns, nutrient intake and diet quality. A study from the international breakfast research initiative group. Nutrients.

[55] Barr S.I., Vatanparast H., Smith J. (2018). Breakfast in Canada: Prevalence of consumption, contribution to nutrient and food group intakes, and variability across tertiles of daily diet quality. A study from the international breakfast research initiative. Nutrients.

[56] Chaumontet C., Even P.C., Schwarz J., Simonin-Foucault A., Piedcoq J., Fromentin G., Azzout-Marniche D., Tome D. (2015). High dietary protein decreases fat deposition induced by high-fat and high-sucrose diet in rats. Br. J. Nutr..

[57] French W.W., Dridi S., Shouse S.A., Wu H., Hawley A., Lee S.O., Gu X., Baum J.I. (2017). A High-protein diet reduces weight gain, decreases food intake, decreases liver fat deposition, and improves markers of muscle metabolism in obese zucker Rats. Nutrients.

[58] Wang L., Du S., Wang H., Popkin B. (2018). Influence of dietary fat intake on bodyweight and risk of obesity among Chinese adults, 1991–2015: A prospective cohort study. Lancet.

[59] Harland J.I., Garton L.E. (2008). Whole-grain intake as a marker of healthy body weight and adiposity. Public Health Nutr..

[60] Wang J., Shang L., Light K., O′Loughlin J., Paradis G., Gray-Donald K. (2015). Associations between added sugar (solid vs. liquid) intakes, diet quality, and adiposity indicators in Canadian children. Appl. Physiol. Nutr. Metab..

[61] Bigornia S.J., LaValley M.P., Noel S.E., Moore L.L., Ness A.R., Newby P.K. (2015). Sugar-sweetened beverage consumption and central and total adiposity in older children: A prospective study accounting for dietary reporting errors. Public Health Nutr..

[62] Laverty A.A., Magee L., Monteiro C.A., Saxena S., Millett C. (2015). Sugar and artificially sweetened beverage consumption and adiposity changes: National longitudinal study. Int. J. Behav. Nutr. Phys. Act..

[63] Seferidi P., Millett C., Laverty A.A. (2018). Sweetened beverage intake in association to energy and sugar consumption and cardiometabolic markers in children. Pediatr. Obes..

[64] Mattes R. (2005). Soup and satiety. Physiol. Behav..

[65] Bolton R.P., Heaton K.W., Burroughs L.F. (1981). The role of dietary fiber in satiety, glucose, and insulin: Studies with fruit and fruit juice. Am. J. Clin. Nutr..

[66] Flood-Obbagy J.E., Rolls B.J. (2009). The effect of fruit in different forms on energy intake and satiety at a meal. Appetite.

[67] US Department of Agriculture Percentages of Selected Nutrients Contributed by Food and Beverages Consumed at Breakfast, by Race/Ethnicity and Age. What We Eat. In America NHANES 2015–2016. https://www.ars.usda.gov/ARSUserFiles/80400530/pdf/1516/Table_14_BRK_RAC_15.pdf.

[68] US. Department of Health and Human Services, U.S. Department of Agriculture (2015). Dietary Guidelines for Americans, 2015–2020.

[69] Lennerz B., Lennerz J.K. (2018). Food Addiction, High-Glycemic-Index Carbohydrates, and Obesity. Clin. Chem..

[70] Filgueiras A.R., Pires de Almeida V.B., Koch Nogueira P.C., Alvares Domene S.M., Eduardo da Silva C., Sesso R., Sawaya A.L. (2019). Exploring the consumption of ultra-processed foods and its association with food addiction in overweight children. Appetite.

[71] Afeiche M.C., Taillie L.S., Hopkins S., Eldridge A.L., Popkin B.M. (2017). Breakfast dietary patterns among mexican children are related to total-day diet quality. J. Nutr..

[72] US Department of Agriculture, A.R.S. Distribution of Meal Patterns and Snack Occasions, by Race/Ethnicity and Age. What We Eat. In America NHANES 2015–2016. https://www.ars.usda.gov/ARSUserFiles/80400530/pdf/1516/Table_34_DMP_RAC_15.pdf.

